# Fundamental Values in Nursing Care: The Person’s Perspective

**DOI:** 10.1177/00469580261427656

**Published:** 2026-06-30

**Authors:** Ivo Cristiano Soares Paiva, Cláudia Patrícia da Costa Brás, Marília Maria Andrade Marques da Conceição e Neves, Rogério Manuel Clemente Rodrigues, Rosa Cristina Correia Lopes, Isabel Maria Pinheiro Borges Moreira

**Affiliations:** 1 University of Coimbra, Portugal

**Keywords:** patient-centered care, nursing care, patient values, professional-patient relations, ethics, professional

## Abstract

Person-centered care requires nurses to integrate the values of people receiving care into nursing practice to promote ethical, humanized care and improved therapeutic outcomes. Despite its relevance, limited evidence identifies which values people themselves consider fundamental and how these values are perceived as integrated into nursing care. In this study, values are understood broadly, encompassing ethical, relational, and professional dimensions. This study aimed to identify the fundamental values of people receiving care, the values they expect from nurses, and the perceived factors influencing the integration of these values into nursing care. The research question was: which values do people consider fundamental for integration into nursing care, and which factors influence this process? A qualitative, descriptive, exploratory study was conducted following the Consolidated Criteria for Reporting Qualitative Research. The study included a convenience sample of 11 participants hospitalized within the previous 6 months. Data were collected through semi-structured interviews, audio-recorded and transcribed verbatim. Content analysis followed Mayring’s approach, including systematic coding, categorization, and thematic abstraction. Ethical approval was obtained, informed consent was secured, and anonymity was guaranteed. Four interrelated themes emerged. These addressed fundamental personal values, values expected from nurses, nurses’ attributes facilitating therapeutic relationships, and perceived organizational and individual factors influencing value integration. Core values emphasized dignity, respect, empathy, privacy, competence, honesty, beneficence, affection, and humanization. Influencing factors included resource constraints, workload, professional awareness, training, vocation, and institutional philosophy. Integrating people’s values into nursing care is essential for ethical, humanized, person-centered practice. Effective integration depends on nurses’ commitment and supportive organizational conditions, including resources, leadership, and alignment with person-centered principles. Future research should examine nurses’ perspectives and organizational strategies.

## Introduction

Understanding the person’s values is essential to nursing care, as values constitute an axiological foundation of human behavior.^1^ Value systems guide interpersonal interactions, influence choices among alternatives, and support decision-making.^1,2^ Active participation of people receiving care requires nurses to understand individuals’ perspectives, values, and preferences.^3^ However, contemporary healthcare contexts are increasingly complex. Cultural diversity, greater awareness of individual rights, technological advances, and social change have made the identification and integration of personal values into care more challenging.^4^

Recognizing people’s values contributes to greater involvement in care and increased satisfaction with nursing care.^5,6^ Despite this, healthcare systems often operate under financial and organizational constraints that prioritize standardized outcomes, potentially limiting the integration of personal values into clinical practice.^7^ Although person-centered care is widely endorsed, there remains a lack of empirical evidence regarding which values people themselves consider fundamental and how they perceive their incorporation into nursing care, constituting a clear gap in the literature.

In this study, values are understood broadly, encompassing ethical, relational, and professional dimensions. This study aims to identify the fundamental values of people receiving care, the values they expect nurses to demonstrate, and the perceived reasons for the integration or non-integration of these values into the care process.

## Background

Individual values vary according to educational, social, demographic, and cultural factors. Although values are personal and hierarchically organized within each individual’s axiological system, research indicates the existence of a relatively stable set of values shared across cultures and countries.^6^ Values are fundamental beliefs that guide attitudes and actions; Kluckhohn defines them as implicit or explicit conceptions of what is considered desirable among available modes, means, and ends of action.^8^

The incorporation of a person’s fundamental and ethical values into clinical practice is essential for achieving person-centered care and sustaining high standards of relational and professional interactions.^1,6,9^ Respecting people’s values, preferences, and needs, and involving them in decision-making, acknowledges each individual as unique and strengthens the therapeutic relationship.^10^ From the person’s perspective, values such as dignity, respect, compassion, empathy, honesty, and open communication emerge as central to meaningful nurse–person interactions.

Providing care requires nurses to harmonize personal values with professional and ethical values, supporting ethical decision-making while respecting individuality and ensuring care quality. Nursing professional values are grounded in the profession’s humanistic and spiritual orientation toward caring and include dignity, compassion, altruism, responsibility, commitment, justice, honesty, and personal and professional competence.^5^ These values provide a framework for evaluating nursing practice and guiding professional behavior.

Nurses’ professional values underpin clinical behaviors, relationships, and decision-making, contributing to professional identity, care quality, job satisfaction, and organizational commitment.^1,2^ Differences in nurses’ professional values across countries relate primarily to prioritization rather than their intrinsic nature.^2^ Nevertheless, increasing health needs, technological complexity, and financial constraints within healthcare systems affect nurses’ ability to integrate personal values into care.^7,9^ The integration of people’s fundamental values has been associated with improved resource use, healthcare practices, continuity of care, and treatment adherence,^5,6^ reinforcing the need to examine these values from the perspective of those receiving care.

## Methodology

### Study Design

A qualitative descriptive and exploratory study was conducted, guided by the Consolidated Criteria for Reporting Qualitative Research (COREQ as Supplemental Material) checklist.^11^ This design was chosen to enable an in-depth exploration of participants’ experiences, perceptions, and meanings related to values in nursing care.

### Participants and Sampling

The sample consisted of 11 participants (7 men and 4 women), aged between 37 and 72 years, recruited through convenience sampling. The inclusion criterion was having been hospitalized within the previous 6 months, ensuring recent experience with nursing care. Exclusion criteria included cognitive impairment or communication difficulties that could compromise participation in the interview.

The sample size was not predefined; participants were recruited until data saturation was achieved, defined as the point at which successive interviews no longer generated new information relevant to the study aims. Data saturation was reached after the 11th interview. There was no prior relationship between the researcher and the participants before study recruitment, and no participants who were invited to take part in the study declined participation or withdrew after consenting.

### Data Collection

Data were collected through individual, face-to-face semi-structured interviews conducted between July and September 2024. The interviews were conducted by a nurse researcher with academic training in nursing and qualitative research.

Interviews took place in a quiet and private setting during working hours, at a time convenient for participants, and each interview lasted approximately 45 min.

All interviews were audio-recorded with participants’ consent and transcribed verbatim. Audio files were securely stored and used exclusively for research purposes. The interview guide was previously validated and comprised open-ended questions designed to explore individuals’ fundamental values, their expectations regarding nurses, and their perceptions of how these values were integrated into nursing care.

No third parties were present during the interviews. Repeat interviews were not conducted, and field notes were not systematically collected during or after the interviews.

### Data Analysis

Data analysis followed Mayring’s qualitative content analysis approach,^12^ using a systematic and interpretative framework. Transcripts were first subjected to a “clean read,” preserving the original meaning while ensuring textual clarity.

Subsequently, latent content analysis was applied to identify underlying meanings, patterns, and relationships among categories. The analytical process involved coding, categorization, and thematic abstraction, with categories continuously reviewed and refined after each interview to ensure methodological rigor and analytical depth.^13^

To avoid interpretative overlap between categorical and latent content analysis, the analytical process was conducted in sequential and complementary phases. Initially, a deductive–inductive categorical analysis was performed to identify manifest content and organize data into clearly defined categories. Subsequently, latent analysis was applied within each stabilized category to explore underlying meanings, relationships, and contextual nuances, rather than generating new categories. This 2-step process ensured conceptual depth while maintaining analytical coherence and grounding latent interpretations in participants’ explicit narratives.

Data coding and analysis were conducted by multiple researchers, with regular meetings held to discuss coding decisions and ensure analytical consistency. Data analysis was conducted manually, without the use of qualitative data analysis software.

### Study Credibility

Study credibility was ensured through strategies aligned with qualitative rigor criteria, namely credibility, transferability, dependability, and confirmability.^14^ Credibility was enhanced through purposive participant selection and achievement of data saturation. Transferability was supported by thick description of participants’ experiences.^15^

Dependability was strengthened through peer debriefing with a health professional knowledgeable about qualitative research and the study topic.^16^ Confirmability was ensured through a transparent description of the analytical process and the inclusion of interview excerpts to illustrate emerging categories. Participants were identified using the letter “I” followed by a number (eg, I1).

To minimize potential researcher bias, reflexive attention was maintained throughout data collection and analysis, ensuring that interpretations remained grounded in participants’ narratives. Interview transcripts were not returned to participants; however, participant validation of the study findings was undertaken by sharing the preliminary results and themes for confirmation.

### Ethical Considerations

This study was approved by the Ethics Committee of the Health Sciences Research Unit: Nursing (UICISA: E) under opinion P1033_04_2024 on May 22nd, 2024.

Confidentiality and anonymity of the data collected were ensured for all participants. Data were used exclusively within the scope of this research and destroyed after transcription.

Participants received an invitation letter and were subsequently contacted. Interviews were scheduled for those who agreed to participate. Participants were asked to sign an informed consent form (explaining the objectives and methodology of the study) and informed that participation was voluntary and anonymous, ensuring confidentiality throughout the study. The informed consent also explained the conditions for participation and included the request for audio recording, in accordance with ethical principles.^16^ After being anonymized, the transcripts were stored by the lead researcher until publication.

## Results

### Sociodemographic Characterization of the Participants

The sample consisted of 11 participants (Table 1), with a mean age of 55.5 years (standard deviation = 7.89 years). Most participants were men (63.64%) and married (81.82%).

**Table 1.** Sociodemographic Characterization of Participants (n = 11).

Attributes	Mean	SD
Age	55.5	7.89
		n (%)
Gender	Male	7 (63.64)
	Female	4 (36.36)
Marital status	Married	10 (81.82)
	Divorced	1 (9.09)
Education	4th grade	1 (9.09)
	9th grade	2 (18.18)
	12th grade	2 (18.18)
	Bachelor’s degree	2 (18.18)
	Master’s degree	2 (18.18)
	PhD degree	2 (18.18)
Occupation	Intellectual and scientific professionals	5 (45.46)
	Technicians and associate professionals	2 (18.18)
	Service and sales workers	1 (9.09)
	Elementary occupations	2 (18.18)
	Retired	1 (9.09)

Regarding education, half of the participants had completed first through ninth grade, while the other half had completed higher education (bachelor’s, master’s, or doctoral degrees).

In terms of occupation, 45.46% were professionals specializing in intellectual and scientific fields, 18.18% were technicians and associate professionals, and 18.18% held elementary occupations.

Four themes emerged from the data analysis: Person’s Fundamental Values, Values Expected from Nurses, Attributes Desired in Nurses, and Perceived Reasons for the Integration or Non-Integration of the Person’s Values into the Care Process (Table 2).

**Table 2.** Themes and Categories.

Themes	Categories
1. Person’s fundamental values	Respect
	Empathy
	Privacy
	Competence
	Honesty
	Interpersonal relationship
2. Values expected from nurses	Respect for the person
	Empathy
	Beneficence
	Honesty
	Affection
	Humanization
3. Attributes desired in nurses	Friendliness
	Availability
	Sense of humor
	Attentiveness
	Effective communication
4. Perceived reasons for the integration or non-integration of the person’s values into the care process	Organizational context
	Limited resources
	Work overload
	Awareness
	Training
	Vocation
	Institutional philosophy

Although the concepts of person’s fundamental values, values expected from nurses, and attributes desired in nurses may appear to overlap, they represent analytically distinct themes in this study. Person’s fundamental values refer to the core beliefs that individuals consider essential to their identity, dignity, and sense of self. Values expected from nurses correspond to the ethical and professional values that participants believe should guide nurses’ conduct throughout the care process. Attributes desired in nurses relate to personal and relational characteristics perceived as facilitating a positive therapeutic relationship. This conceptual distinction guided the thematic analysis and the organization of the findings.

### Theme 1: Person’s Fundamental Values

Participants identified the following as **fundamental values**: respect, empathy, privacy, competence, honesty, and interpersonal relationships.

**Respect** was the value most frequently mentioned by participants during the interviews. Participants considered **respect** for their values to be essential when receiving care. According to the participants, care must acknowledge the uniqueness and vulnerability of each person.

*“I think that values must be respected.”* (I3)

*“To see the person and not the illness. . . to see the person as important. . . to respect them.”* (I9)

**Empathy** was also identified as a fundamental value: the ability to understand others and to recognize their vulnerability, or to be able to put oneself in their position. Participant I5 stated, *“Having empathy for others is knowing how to put yourself in their shoes”*.

Participants’ narratives also described **privacy** as fundamental. They valued situations in which nurses took care to preserve their privacy, such as using screens or curtains to prevent unnecessary exposure during procedures. Participants also highlighted the importance of nurses acknowledging potential embarrassment when people were treated by professionals of the opposite sex and ensuring that such situations were avoided in the future.

*“I think privacy is important.”* (I5)

*“(. . .) They were careful when they came to do hygiene, they always closed the screen, especially because I didn’t feel very comfortable when male nurses, of course(. . .) did this work. I felt a little embarrassed, they must have realized this and from then on (. . .) they respected my privacy.”* (I6)

Participants’ narratives about respect for privacy seemed to encompass not only respect for physical intimacy but also respect for the confidentiality of personal matters.

*“The rooms there aren’t that big either, and I overheard the conversations between nurses or doctors and the patient next to me, there’s no privacy there. (. . .) I think these conversations should take place in the office.”* (I1)

**Competence** was another fundamental value that participants expected nurses to demonstrate. When nurses did not demonstrate competence, participants felt insecure. They mentioned that behaviors such as forgetting a person’s name after interacting with them more than once or taking “*notes on a [random] piece of paper, important notes about the person’s condition. . .I think* that is *not very professional*” (I1) were signs of incompetence. Conversely, when nurses demonstrated their knowledge and skills, the care they provided contributed to the person’s sense of security.

*“I really liked the treatment because you could see that they knew what they were doing.”* (I1)

Participants highlighted **honesty** as a crucial moral quality for nurses. They valued sincerity and transparency when receiving information because it made them feel safe throughout the care process.

*“I admire that, the honesty, respect, empathy, which are fundamental to me, I experienced those aspects.”* (I4)

*“(. . .) If we don’t know very well what’s coming next, we worry. . . we always have that feeling. . . did something go wrong?”* (I3)

Participants also emphasized the importance of the **interpersonal relationship** established with nurses. They highlighted that nursing care should extend beyond technical procedures; nurses’ interaction and dialog with patients were seen as key to making them feel at ease and less alone and isolated. Furthermore, this contributes to a less distressing hospitalization experience.

*“(. . .) Nurses have to be more attentive to talking, it’s not just about doing the treatment. . . let’s say it facilitates the hospitalization process much, much more, because some people feel isolated there (. . .)”* (I6)

*“Because (. . .) they also asked where I was from, if I had children. They asked more personal questions to put me at ease”* (I8)

### Theme 2: Values Expected From Nurses

Participants emphasized that nurses must embody certain values because, without them, they cannot be considered good professionals, given that nursing is a profession centered on caring for people.

*“Being a nurse requires certain values (. . .) no matter how hard they try, they will never be good nurses, they have to have values!”* (I1)

*“Those who work in health and care have a very strong human side. They are not machines. . .”* (I3)

According to the participants, the values expected from nurses are: respect for the person, empathy, beneficence, honesty, affection, and humanization.

**Respect for the person** was recognized as a value expected from nurses, considering that every person is different. Respect for the person encompasses not only the physical dimension but also a person’s convictions and decisions. When professionals fail to ask what matters to a person or disregard their decisions, that person feels objectified, and a fiduciary relationship is not established between them and their nurse.

*“You have to respect them in the sense of respecting their differences.”* (I4)

*“Treat people with respect, and consideration for their physical and mental state, which is very, very fragile (. . .)”* (I7)

*“Nobody asked what I felt, what I wanted, (. . .) how I was living the moment, (. . .) so I couldn’t feel that relationship of trust. I felt like another object (. . .) professionals, in this case nurses, didn’t respect me as a person.”* (I2)

**Empathy** was described as a priority value that people receiving care expect nurses to demonstrate, particularly since they are vulnerable when seeking healthcare. Participants expected nurses to be sensitive to their problems and able to empathize with them.

*“But empathy is fundamental because people who go to the hospital, like me a few times, are sick and vulnerable, and nurses are expected to provide it.”* (I4)

*“Empathy, trying to put yourself in my place, what I’m feeling”* (I2)

**Beneficence** was also highlighted. Participants expected nurses to demonstrate dedication, that *“the nurse is doing the best for the patient”* (I4), thus ensuring their professionalism and commitment to well-being.

*“The vein was already [showing signs of phlebitis] and she was aware of this. That was very important to me.”* (I5)

*“Doing the best for the patient. I think it’s fundamental.”* (I4)

**Honesty** was another essential value in nurses’ interactions. Participants expected nurses to be truthful and valued honesty in all situations. They also expected nurses to acknowledge mistakes, recognizing that everyone makes mistakes. What mattered most to participants was that nurses admit their mistakes without making excuses or shifting blame.

*“Be honest, be nice, try, but be truthful with people”* (I1)

*“I felt that the professionals were honest with me. The first time, for example, they were honest, they told me, this didn’t go well.”* (I4)

*“Because everyone makes mistakes, we have to acknowledge them, we can’t dismiss, ignore them, or make excuses”* (I1)

**Affection** was another value that participants expected to see in nursing practice. They stressed that sick people are vulnerable and that nurses should demonstrate affection rather than focus solely on technical procedures.

*“But those who are hospitalized are vulnerable, regardless of the situation. No matter how annoying a patient is, they should make an effort to show affection.”* (I5)

**Humanization** of care was also emphasized as a guiding principle in care. Participants argued that nurses should focus on the person during care delivery, showing genuine interest in their well-being and recovery. Humanized care helps sick people who feel fragile and vulnerable feel comforted and not abandoned.

*“During the hospitalization, they should treat people well, if they treat people with humanity, make them feel as comfortable as possible, because I felt fragile, I felt completely abandoned (. . .)”* (I7)

*“This human side, this genuine interest they showed me are very important values.”* (I3)

In their narratives, participants asserted that nurses should focus not only on technical tasks but also on the human dimension of the person. They mentioned additional care aspects that cannot be translated yet are fundamental, such as “*Ensuring a reassuring presence*” (I4), addressing people by their name, and respecting their convictions.

*“(. . .) To go a little bit beyond the technical aspects. They respect my way of being. My way of life. Addressing the person by their name is something I think is very important.”* (I4)

### Theme 3: Attributes Desired in Nurses

Participants identified several **attributes** they **desired in** nurses: friendliness, availability, sense of humor, attentiveness, and effective communication.

For interviewees, these attributes were essential for building a relationship between the nurse and the person receiving care. **Friendliness** was highlighted as a facilitator of interaction with others. According to the participants, people value being cared for by someone who smiles and speaks calmly, noting that such attitudes made a difference in their experience of illness.

*“Oh, it was the way they talked to me. . . all friendly. And their smile, they smiled. It’s important for someone in this situation (. . .) to see a smile (. . .) a calmer tone of voice and this make a difference to someone who is sick.”* (I9)

**Availability** was also valued as a primary attribute for nurses. Participants emphasized that nurses should rethink their care practices and adopt an attitude of active listening, openness, and receptivity to the other person while delivering care.

*“Sometimes they come, cross their arms, wait for the answer, and go away, so being available to listen to us is very important.”* (I2)

*“Being available to listen to the person, to listen to their problems, and that was done. I think that’s the first thing. . .”* (I1)

**Sense of humor** was described by participants as a facilitator of communication because it helps ease tension in difficult or unpleasant situations. When nurses use humor with people they already know, it can serve as a way of meeting their needs.

*“I think they must also have a sense of humor, to break the ice”* (I3)

*“[They] played with the situation and I think this is a way of trying to reach us, to show that they know us.”* (I5)

**Attentiveness** was another attribute that participants expected from nurses, given the fragile and vulnerable state of people who are sick.

*“I think they should be, they should be attentive because people are fragile. So, give attention, be attentive.”* (I5)

Participants also valued nurses’ **effective communication** or interaction with people receiving care, noting that the use of both verbal and non-verbal communication is fundamental and makes a meaningful difference for those who are sick.

*“To come, to greet, to smile, which are small things, but they make a lot of difference.”* (I5)

*“You can tell by the language and sometimes by the gestures. We can tell right away from the way they do things and in their tone of voice.”* (I8)

For participants, careful communication by nurses fosters the empowerment of people receiving care and facilitates the negotiation of care.

*“(. . .) Look, I’m going to do this and that, if it hurts, tell me and I’ll try to fix it (. . .). Let’s do it this way, they negotiated with me some types of care”* (I7)

Theme 4: Perceived Reasons for the Integration or Non-Integration of the Person’s Values in the Care Process

Participants reflected on the perceived **reasons** for the **integration or non-integration of the person’s values in the care process**, identifying both facilitating and hindering factors. Facilitating factors included awareness, training, vocation, and institutional philosophy, whereas hindering factors were associated with the organizational context, limited resources, and work overload.

According to participants, nurses must understand the importance of integrating the person’s values into care. This requires actively asking the person receiving care about what matters to them because values are fundamental to each individual. Participants believe that **awareness** involves both professional training in this field and reflection on practices.

“*First, I think they need to understand that values are important and that there are values that are important to each person, and that they must integrate them every day into the care they provide. Maybe they need to pause and reflect, perhaps they also need training on this and should ask people questions*” (I2)

Professional **training** was identified as a key facilitator of the integration of the person’s values into the care process. Participants reported that nurses should develop a socio-moral capacity that aligns with their professional practice and considered training in the field of values to be fundamental. They emphasized that values must be integrated into nursing education.

*“I think many complaints about healthcare, especially concerning nurses, are related to the lack of respect people feel when they use the services, and that is something you learn, you learn it, but it must be taught.”* (I4)

*“These values need to be instilled during classes, at school; many students already come here with an arrogant attitude.”* (I7)

Participants also discussed the importance of **vocation** for being a nurse in order to adequately meet people’s needs.

“*I think if you want to be a nurse, you need to have a passion for it, it’s a vocation. But some have it, and others don’t!”* (I8)

The **institutional philosophy** was described as a factor that facilitates the integration of the person’s values into the care process. Participants perceived a difference in the respect shown for their values between private and public institutions. They felt that professionals in the private sector were more concerned with them as individuals and not solely focused on their clinical situation.

*“In public services, it’s more difficult to create this type of environment”* (I3)

*“I think that they were more concerned with me because it was a private unit. Not just concerned with the situation. But I don’t know if it’s the same or not in a public hospital.”* (I8)

The **organizational context** of healthcare institutions refers to their structure and the fact that multiple hospitalized patients may share the same physical space at the same time, along with the presence of several nurses. This means that conversations between professionals and people receiving care, as well as between people and their families, often occur without respect for individual privacy. Participants state that adhering to service routines results in their needs and choices not being respected, making them feel disrespected as individuals.

*“The space, the resources (. . .) the lack of conditions.”* (I5)

*“the routines of the service, which were imposed, ignoring my needs and choices, and because of this, I felt very vulnerable, fragile, and really insignificant. (. . .) I was not respected as a person.”* (I4)

Institutions’ **limited resources**, whether technical, human, structural, or time-related, were also seen as unfavorable conditions for integrating the person’s values. Participants also considered “*the number of patients under the care of each health professional*” (I11) an obstacle to the integration of the person’s values into the care process.

Participants further noted that **work overload** puts nurses under constant stress, which often compromises both their own values and those of people under their care. This pressure could lead to indifference and a disregard for people’s individuality. Participants also believed that nurses’ values are influenced by the pressure of work in a stressful environment.

*“neutral attitude, a bit like going through the motions, where things just aren’t valued”* (I4)

*“They may have values, but working in stressful situations sometimes causes them to set those values aside because they are anxious, they are tense.”* (I5)

However, some participants argued that workload alone was not sufficient to explain the non-integration of the person’s values into the care process. They suggested that, even during care procedures, nurses could take time to interact with people, assess their needs, values, and preferences, and incorporate them into care. In their view, failing to do so was more closely related to professional competence than to work overload.

*“Many times, when the nurse administered the antibiotic, they could have taken that moment to talk to me, introduce themselves, ask how I was feeling? They need to rethink how they approach us and how they can meet our needs, choices, and values, the values of the clients.”* (I2)

## Discussion

This study revealed a strong congruence between the person’s fundamental values and the values expected from nurses. This finding reinforces the central premise of person-centered care, namely that ethical and relational principles should be shared and enacted by all actors involved in the care process. Participants emphasized values such as respect, empathy, privacy, competence, honesty, and interpersonal relationships.

This alignment is consistent with established person-centered care frameworks that highlight dignity, autonomy, partnership, and responsiveness as core dimensions of quality care.^6,9^

These findings are consistent with seminal theoretical models of person-centered nursing. McCormack and McCance’s Person-Centered Nursing Framework emphasizes the integration of personal values, professional competence, and care processes that honor the person’s individuality and lived experience.^17^ Similarly, Kitson et al’s conceptualization of patient-centered care underscores the importance of shared values, meaningful relationships, and contextual enablers to achieve person-centered outcomes.^18^ The congruence identified in this study supports these frameworks by demonstrating that people receiving care recognize and value the same ethical principles advocated in nursing theory.

Respect emerged as the most salient value, understood as recognition of the person’s uniqueness, dignity, and human rights.^19^ This finding corroborates previous research identifying respect as a foundational element of person-centered nursing, enabling individualized responses to physical, emotional, and psychosocial needs while preserving autonomy and integrity.^6,20,21^ Empathy and privacy were also highlighted as essential values. Empathy facilitates therapeutic relationships and inclusive care, while privacy promotes trust, dignity, and satisfaction with care.^6,22,23^ These results are consistent with existing literature and reinforce the ethical and relational dimensions of nursing practice.

Competence and honesty were identified as structuring values that support safety, credibility, and shared decision-making.^23^ While prior studies emphasize technical competence as central to care quality, participants in this study also highlighted relational competence, suggesting that professional expertise alone is insufficient without transparency and ethical consistency.^9^ This nuance contributes to existing knowledge by reinforcing competence as an integrative construct encompassing both technical-scientific and relational skills.

Recent international evidence reinforces the relevance of these findings within a global context of person-centered healthcare. Studies conducted across diverse healthcare systems have consistently shown that respect for personhood, relational continuity, and the active integration of people’s values are central determinants of care quality, safety, and trust in healthcare professionals.^24,25^

In particular, international nursing and health services research highlights that person-centered care contributes to improved experiences of care, greater ethical sensitivity in clinical practice, and more sustainable health systems.^24-26^

Recent empirical and conceptual work further emphasizes that the integration of people’s values into care is not solely dependent on individual professionals, but is strongly shaped by organizational cultures, leadership, and system-level priorities.^25,26^

International authors argue that without supportive structures, manageable workloads, and explicit institutional commitment, the enactment of person-centered values remains fragmented and inconsistent, regardless of professionals’ ethical intentions.^24,26^

These global perspectives strengthen the transferability of the present findings and underscore the need for organizational- and policy-level strategies to support value-based nursing practice.

In addition to values, participants identified attributes such as friendliness, availability, attentiveness, sense of humor, and effective communication as facilitators of therapeutic relationships.^27^ Although these attributes may appear to overlap with values, they are better understood as relational expressions through which values are enacted in practice. This distinction clarifies previous ambiguities in the literature and highlights how values are operationalized in everyday interactions.^28-30^

Despite the recognized importance of values and attributes, participants reported several organizational barriers. These included workload, limited resources, and rigid routines that hinder the consistent integration of values into practice.^28,31,32^ This evidence aligns with previous research showing that task-oriented environments often marginalize relational care, contradicting person-centered ideals. This discrepancy underscores the need for organizational and policy-level interventions.

To address these challenges, organizational strategies should include the integration of value-based indicators into quality assessment frameworks, the allocation of protected time for relational care, and leadership models that explicitly prioritize person-centered values. Structured ethics reflection forums, interdisciplinary case discussions, and continuous professional development programs focused on ethical sensitivity and communication may further support the integration of people’s values into everyday practice. Additionally, institutional policies that promote continuity of care and reduce task fragmentation can facilitate meaningful nurse-person relationships.

From an ethical perspective, the findings indicate that the non-integration of people’s fundamental values constitutes not only a relational failure, but also an ethical concern with implications for dignity, autonomy, and justice. Situations in which individuals feel objectified, unheard, or exposed reflect ethical vulnerabilities that challenge core nursing values and professional ethical codes. The results suggest that ethical practice in nursing extends beyond individual moral intent and requires organizational conditions that enable ethical sensitivity, moral agency, and value-based decision-making.

The findings have important implications for nursing practice, education, and policy. Integrating values-based indicators into quality assessment, strengthening ethics and communication training, and fostering supportive organizational cultures are essential strategies to promote person-centered care.^28,33^ Future research should explore nurses’ perspectives across diverse cultural contexts and examine organizational strategies that enable sustainable value-based practice.

A key limitation of this study is the cultural and ethnic homogeneity of the sample, which limits the transferability of findings. Values related to care are culturally embedded, and the absence of diverse perspectives restricts intercultural sensitivity. Future studies should include culturally diverse populations to better understand how values, spirituality, family roles, and autonomy intersect in person-centered nursing care.

## Conclusion

This study highlights that integrating people’s fundamental values into nursing care is central to delivering ethical, humanized, and genuinely person-centered care. The findings demonstrate a clear alignment between the values individuals consider essential (such as respect, empathy, privacy, competence, honesty, and meaningful interpersonal relationships) and the values they expect nurses to enact in practice. This convergence reinforces the relevance of person-centered care frameworks and underscores the role of shared ethical and relational principles in enhancing care quality, safety, and satisfaction for both people receiving care and nurses.

These findings reinforce that ethical nursing practice cannot be sustained solely through individual professional commitment. Healthcare organizations and systems bear ethical responsibility for creating conditions that enable the consistent enactment of person-centered values, safeguarding dignity, respect, and moral integrity in care delivery. The study also demonstrates that values alone are insufficient without conditions that enable their enactment. Participants identified organizational and structural constraints, including workload pressures, limited human and material resources, and rigid institutional routines, as major barriers to the consistent integration of values into everyday nursing practice. These findings emphasize that responsibility for person-centered care extends beyond individual professionals to healthcare organizations and systems.

Importantly, the results suggest that ethical, relational, and technical competence must be developed in an integrated manner. Continuous professional education, reflective practice, and organizational cultures that explicitly prioritize person-centered values are essential to bridging the gap between theoretical principles and clinical reality.

Although the study provides valuable insights into the person’s perspective on values in care, the cultural and ethnic homogeneity of the sample limits the transferability of the findings. Future research should therefore include more diverse populations and explore organizational strategies that support sustainable, value-based nursing practice across different cultural and care contexts.

## Supplemental Material

sj-docx-1-inq-10.1177_00469580261427656 – Supplemental material for Fundamental Values in Nursing Care: The Person’s Perspective Supplemental material, sj-docx-1-inq-10.1177_00469580261427656 for Fundamental Values in Nursing Care: The Person’s Perspective by Ivo Cristiano Soares Paiva, Cláudia Patrícia da Costa Brás, Marília Maria Andrade Marques da Conceição e Neves, Rogério Manuel Clemente Rodrigues, Rosa Cristina Correia Lopes and Isabel Maria Pinheiro Borges Moreira in INQUIRY: The Journal of Health Care Organization, Provision, and Financing

sj-docx-2-inq-10.1177_00469580261427656 – Supplemental material for Fundamental Values in Nursing Care: The Person’s Perspective Supplemental material, sj-docx-2-inq-10.1177_00469580261427656 for Fundamental Values in Nursing Care: The Person’s Perspective by Ivo Cristiano Soares Paiva, Cláudia Patrícia da Costa Brás, Marília Maria Andrade Marques da Conceição e Neves, Rogério Manuel Clemente Rodrigues, Rosa Cristina Correia Lopes and Isabel Maria Pinheiro Borges Moreira in INQUIRY: The Journal of Health Care Organization, Provision, and Financing
