# Supplementary material for: Fundamental Values in Nursing Care: The Person’s Perspective
Source: Inquiry. 2026 Jun 30;63:00469580261427656. doi: 10.1177/00469580261427656 (PMC13319766; doi:10.1177/00469580261427656)
Supplement: sj-docx-2-inq-10.1177_00469580261427656 – Supplemental material for Fundamental Values in Nursing Care: The Person’s Perspective [file sj-docx-2-inq-10.1177_00469580261427656.docx]

**Interview guide – Values**

| **Location:** To be determined according to the participant. | | |  |  |
| --- | --- | --- | --- | --- |
| **Interviewer:** Members of the Ethics Disciplinary Group | | |  |  |
| **Theme: Prioritized values of the person in the care process**  **Significant value for the person when cared for.** | | |  |  |
| **Objectives:** To obtain data on:  **-** the fundamental values for the person being cared for.  - whether the person's values are integrated into the caregiving process  - how the person's values were integrated into the caregiving process.  - the reasons perceived by the person for not integrating their values into the caregiving process | | |  |  |
| **Interview #_____** | | |  |  |
| **Date:___/___/___** | | |  |  |
| **Start time:_______ End time:_______** | | |  |  |
| **LEGITIMATION OF THE INTERVIEW** | | | |  |
| **Motives and intentions of the investigator** | 1. Presentation of the study 2. Explanation of the interview:  - Objectives - What is expected of the interviewee and the reason for their selection. - Interviewer's role - Estimated duration - Data recording - Data analysis/negotiating strategies for validating data analysis.  1. Provide information on how to ensure the anonymity of the interviewee. 2. Inform them about the possibility of interrupting the interview. 3. Request written authorization to conduct the interview. 4. Request written permission for audio recording of the interview. | ***Observations***  Combining strategies for validating information | |  |
| **Characterization of the person** | 1. Characterization of the person  - Age: _________________________________ - Sex: __________________________________ - Marital status: ____________________________ - Academic qualifications: __________________ - Professional activity: __________________ |  | |  |
| **Narrative Phase** | ***Questions:***  Tell me about the values that are fundamental to you as a person? Points to explore: the meanings you assign to them.  What values did you consider fundamental to be respected when you were cared for during your hospitalization?  During the care process, did the nurses ask you what was important to you?  What aspects do you think interfered with the valuation and integration of your values into the care process?  What values and principles do you believe nurses should uphold when caring for a person?  What values did the nurse consider important to incorporate when caring for you?  - What does it mean (or did it mean) to you when the nurse values your worth and integrates you into the care process?  - In your understanding, what factors positively influenced the integration of your personal values into the care process during hospitalization?  - What obstacles do you think have existed in healthcare services that hinder the integration of the values of sick people during care?  How can nurses improve/facilitate the integration of their core personal values into the care process during their final hospitalization? | ***Observations***  - Adopt an attentive posture and a sensitive attitude that is conducive to storytelling:   - Look at the interviewee's face in a natural and direct way. - Respect the interviewee's silence.   -Request clarification  Can you explain what you meant?  Request examples | | |
| **Balance Phase** | | | |  |
|  | ***Questions:***  **-** During the care process, at what points did you feel that the nurses valued/integrated your values?  **,** what experiences contributed favorably and what hindered the expression of your values? |  | |  |

| **END OF INTERVIEW** |
| --- |
| Are there other aspects of this topic that you would like to add, or that I haven't asked about, and that you would like to discuss?  Summarize the essential aspects covered during the interview.  - Thank the interviewee for their collaboration and mention the importance of their participation in the study  - Negotiate the analysis validation process. |
